# Influence of the Type of Marking and the Number of Players on Physiological and Physical Demands During Sided Games in Soccer

**DOI:** 10.1515/hukin-2015-0081

**Published:** 2015-10-14

**Authors:** David Casamichana, Jaime San Román-Quintana, Julen Castellano, Julio Calleja-González

**Affiliations:** 1 Faculty of Physiotherapy and Speech Therapy Gimbernat-Cantabria University School associated with the University of Cantabria (UC). Torrelavega, Spain.; 2 Faculty of Physical Activity and Sport Sciences. University of the Basque Country (UPV/EHU). Vitoria-Gasteiz, Spain.

**Keywords:** exercise intensity, specific training, time-motion, GPS device, heart rate, man-marking

## Abstract

The aim of this research was to examine the influence of two variables, the type of marking (with or without man-marking) and the number of players per team (3, 6, or 9) on the physical and physiological demands of sided games in soccer. Eighteen amateur players were monitored with GPS and heart rate devices. The following variables were analyzed: a maximum heart rate, a mean heart rate, time spent in each intensity range, total distance covered and distance covered in different speed ranges, a player load, maximum speed reached, and a work:rest ratio. The results showed that the type of marking influenced the physical demands of players, with greater total distance, a player load and a work:rest ratio when man-marking was used in the 3 vs. 3 (737 m, 95 Arbitrary Units (AU) and 3.4 AU, respectively) and 6 vs. 6 (783 m, 95 AU and 5.3 AU, respectively) games (p<0.05). The number of players also had an effect on physiological intensity, with more time being spent at the <80%HRmax during the 9 vs. 9 and 6 vs. 6 games (more than 30%) compared with the 3 vs. 3 format (less than 15%) (p<0.05). These findings could help coaches to understand how the modification of different variables in sided games influences the physical and physiological demands of players.

## Introduction

For a number of decades sided games (SGs) have been used in soccer as an alternative to fitness training without the ball (Allen et al., 1998; Hoff and Helgerud, 2004). The aim of SGs is simultaneously to improve technical, tactical, and physical aspects of players’ performance (Little, 2009) and research has shown that they are as effective as interval training (Hill-Haas, Coutts et al., 2009; Impellizzeri et al., 2006).

Furthermore, coaches can modify the intensity of SGs according to the aims of training (Aguiar et al., 2013), by changing several variables such as the number of players taking part (Brandes et al., 2011; Hill-Haas, Dawson et al., 2009; Köklü et al., 2011), dimensions of the playing area (Aslan, 2013; Casamichana and Castellano, 2010; Kelly and Drust, 2009), rules of the game (Hill-Haas et al., 2010), the use of “floater” players (Mallo and Navarro, 2008), the number of touches (San Román-Quintana et al., 2013), the game format (Castellano et al., 2013) or the number and duration of task repetitions (Casamichana et al., 2013; Fanchini et al., 2011; Hill-Haas, Rowsell et al., 2009).

One of the variables that has been less widely analyzed in the literature is the type of marking used during SGs (Aroso et al., 2004; Ngo et al., 2012; Sampaio et al., 2007). Aroso et al. (2004) compared games with (man-marking; MM) and without man-marking (no man-marking; NMM) and found an increase in blood lactate concentration when MM was used. Sampaio et al. (2007) found that players reported higher ratings of perceived effort (RPE) when MM was used, although there were no significant differences in terms of physiological response. More recently, Ngo et al. (2012) reported that the percentage heart rate reserve (%HRres) was significantly higher in games involving MM, regardless of whether goals were used.

With regard to the influence of the number of players and the relative pitch area available to each player, research generally shows that physiological intensity is higher with fewer participants (Brandes et al., 2011; Dellal et al., 2011; Köklü et al., 2011), although the mean values for distance covered and duration of sprints are reported to be lower than when more players take part. To our knowledge, however, there are no published studies that have simultaneously examined the influence of both these variables, that is, the type of marking and the number of participants, on the physical and physiological demands of amateur soccer players during SGs. Thus present study seeks to address this issue by analyzing whether the defensive rule (MM or NMM) and the number of players per team (SGs of 3 vs. 3, 6 vs. 6, and 9 vs. 9) influence the physical and physiological response of players.

Therefore, the aim of this study was to examine the influence of two variables, the type of marking (with or without man-marking) and the number of players per team (3, 6, or 9), on the physical and physiological demands of sided games in soccer. Based on previous reports, our hypothesis assumed an influence of the two variables on the internal and external load variables.

## Material and Methods

### Participants

Participants were 18 amateur players (age: 23.4 ± 4.5 years; body height: 178.7 ± 5.6 cm; body mass: 74.4 ± 6.1 kg; Yo-Yo intermittent recovery test 1 (YYIRT1): 2438.5 ± 540.3 m) (Bangsbo et al., 2008) who played for the same team at the regional level. They had played federation soccer for a mean of 12.5 years prior to the study. Their standard training involved 3 sessions per week, in addition to a competitive match. All the players were notified of the research design and its requirements, as well as of the potential benefits and risks, and they each gave their informed consent prior to the start. The study was conducted according to the Declaration of Helsinki (2008), and the Ethics Committee of the University of the Basque Country (CEISH) gave institutional approval for the study.

### Procedures

The study was performed over a 6-week period (February-March) during the 2010–2011 competitive season. During week 1 all players performed the YYIRT1 in order to determine their maximum heart rate (HR_max_; Bangsbo et al., 2008). This was followed by 12 training sessions carried out on an artificial grass pitch and at a similar time of day (8:30 pm). A minimum rest period of 48 h was scheduled between sessions. Each session began with a standardized 15-min warm-up, followed by a SG of 6 min duration.

In order to minimize potential imbalances between teams and to ensure their equivalence, the procedure proposed by Casamichana and Castellano (2010) was followed. Active encouragement by coaches was provided in all the SGs in order to ensure high player motivation (Rampinini et al., 2007).

### Measures

#### Independent variables: type of marking and number of players per team

The independent variables were the type of marking and the number of players. The aim of all the SGs was to maintain ball possession for longer than the opponent (there were no goals). The only variables modified were the type of marking (two levels: MM or NMM) and the number of players per team (three levels): 1) 3 *vs.* 3 on a playing area of 19 × 29 m; 2) 6 *vs.* 6 on a playing area of 40 × 28 m; and 3) 9 *vs.* 9 on a playing area of 55 × 30 m. In those games where MM was used each player was assigned an opposing player who they had to mark when their own team was not in possession of the ball. On the other hand, NMM was equivalent to zonal marking. The relative pitch area per player was similar in each of the SGs (~ 92 m^2^).

#### Physiological profile: heart rate

Physiological demands were assessed on the basis of the heart rate (HR) (Esposito et al., 2004), using a telemetric device (Polar Team Sport System, Polar Electro Oy, Finland). In line with previous research, we established six HR intensity zones: <50%HR_max_, 50–60%HR_max_, 60–70%HR_max_, 70–80%HR_max_, 80–90%HR_max_, and >90%HR_max_ (Casamichana, Castellano and Dellal 2013; Hill-Haas, Dawson et al., 2009). Both absolute HR data and relative values with respect to the mean heart rate (HR_mean_) and HR_max_ (i.e., %HR_mean_ and %HR_max_) were analyzed. The percentage of time spent in each intensity zone was also examined.

#### Physical profile: distance covered, speed and accelerations performed

Players were monitored using GPS devices operating at a sampling frequency of 10 Hz (MinimaxX v.4.0, Catapult Innovations). This technology had been shown to produce valid recordings of high-intensity movements in sport (Castellano et al., 2011; Varley et al., 2012).

The variables analyzed were the total distance covered, the distance covered in different speed ranges: 0–6.9, 7.0–12.9, 13.0–17.9, 18.0–20.9 and >21, all in km·h^−1^ (Hill-Haas, Dawson et al., 2009), and the number of accelerations produced in different intensity ranges: 1.0–1.5, 1.5–2.0, 2.0–2.5, and >2.5, all in m^.^s^−2^ (Varley et al., 2012).

Global load indicators were also measured, specifically the work:rest ratio (WR), the maximum speed reached (V_max_), and the player load (PL). The latter is an indicator based on the combined accelerations made in three planes of movement. Research on this indicator had reported high intra- and inter-device reliability (Boyd et al., 2011), and it had been shown to be a valid way of monitoring a training load in soccer players (Casamichana, Castellano, Calleja-González et al., 2013).

### Statistical analysis

The data are presented as means and standard deviations (mean ±SD). The homogeneity of variances was examined by means of the Levene’s test, and the presence of significant differences was determined in two ways: for the variable with two levels (type of marking) we used the Student’s *t* test for independent samples, while the variable with three levels (number of players) was examined by means of a two-way repeated measures analysis of variance (ANOVA), applied to each of the dependent variables. A post hoc Bonferroni test was also applied to make pairwise comparisons between the different levels of within-player factors. Cohen’s *d* Effect sizes (ES) were calculated (Rhea, 2004) for physiological responses and time-motion characteristics. All the statistical analysis were performed using SPSS 16.0 (SPSS Inc.,Illinois, USA) for Windows, with significance being set at *p*<0.05.

## Results

### Physiological profile: heart rate

Table 1 shows absolute mean values (in bpm) for HR_mean_ and HR_max_, as well as percentage mean values for both these measures (i.e., %HR_mean_ and %HR_max_),which were calculated relatively to the maximum individual value obtained on the YYIRT1. Significant differences were observed in relation to the %HR_mean_. When MM was used the %HR_mean_ was significantly higher in the 3 *vs*. 3 SGs compared with the 9 *vs.* 9 SGs (F_(2,27)_=3.56, *p*=0.009; ES=1.06). In the 6 *vs.* 6 SGs the %HR_mean_ was significantly higher when NMM was required (T _(18)_=2.71, *p*=0.008; ES=1.41).

**Figure 1:**
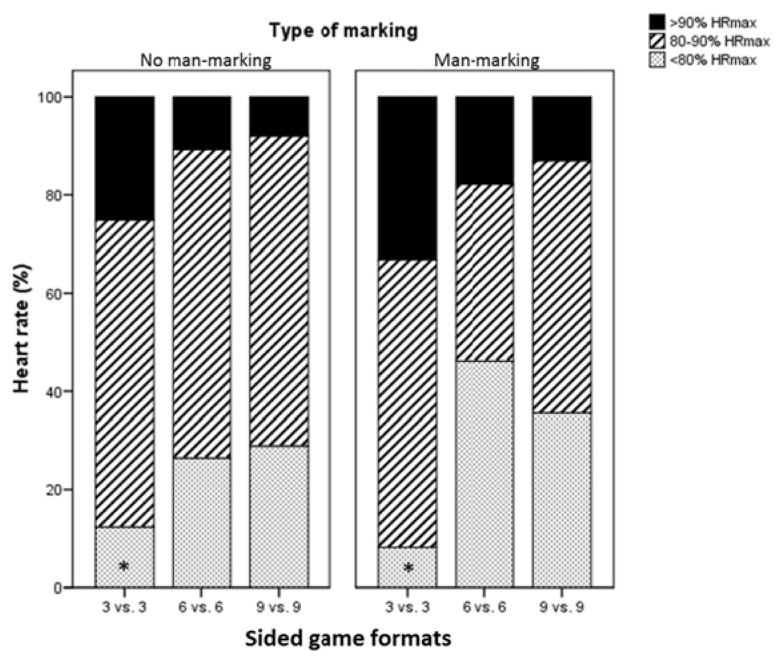
Distribution of the heart rate across intensity zones (%) for each type of SG. * is < 6 vs. 6 and 9 vs. 9.

Figure 1 shows the distribution of the HR (%) across intensity zones for each of the SGs. There were no significant differences associated specifically with either the type of marking or the number of players per team. When the data were pooled and analyzed regardless of the type of marking, the results showed that players performed significantly longer at an intensity <80% HR_max_ in the 6 *vs.* 6 and 9 *vs.* 9 SGs as compared with the 3 *vs.* 3 games (F_(2,49)_=5.44, *p*=0.023; ES=0.83).

### Physical profile: distance covered, speed and accelerations performed

Table 2 shows mean values (±SD) for the distance covered (in m), the player load (in AU), maximum speed achieved (km·h^−1^), and the work:rest ratio (in AU) with respect to the type of marking and the number of players in the SGs. The distance covered differed significantly depending on the number of players, both with respect to NMM SGs (F_(2,25)_=8.66, *p*=0.001; ES=2.00) and in terms of the mean values (F_(2,55)_=5.16, *p*=0.009; ES=0.77–0.94). Finally, the V_max_ differed significantly depending on the number of players for both types of marking (NMM SGs: F_(2,25)_=7.28, *p*=0.003; ES=2.00; MM SGs: F_(2,27)_=5.73, *p*=0.008; ES=1.20), as well as in relation to the mean values (F_(2,55)_=12.81, *p*=0.000; ES=1.54).

The results in Table 2 show that for 3 *vs*. 3 games the type of marking was associated with significant differences in the distance covered (T _(18)_=2,99; *p*=0.008; ES=1.35), the player load (T_(18)_=2,51; *p*=0.022; ES=1.12), and the work:rest ratio (T_(18)_=2,48; *p*=0.023; ES=1.22). In 6 *vs.* 6 games, significant differences related to the defensive rule were only observed for the distance covered (T_(18)_=2,29; *p*=0.034; ES=1.04) and the work:rest ratio (T_(18)_=2,363; *p*=0.035; ES=1.09). The type of marking produced no significant differences in the 9 *vs*. 9 format. However, the type of marking was associated with significant differences in the distance covered when analyzing the mean values obtained (T_(18)_=2,429; *p*=0.018; ES=0.64).

**Figure 2:**
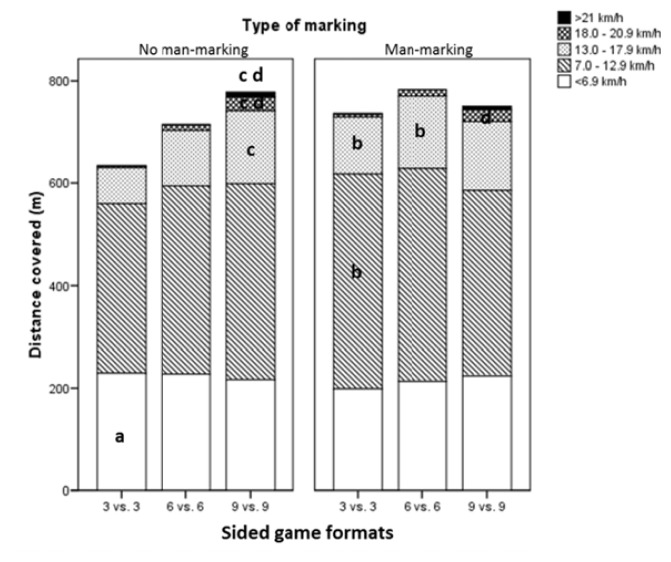
Total distance covered (m) in the established speed ranges for each of the SGs: 3 vs.3, 6 vs. 6, and 9 vs. 9. Note: the Bonferroni post hoc test, with p<0.05 in all cases. ^a^ is > man marking, ^b^ is > no man marking, ^c^ is > 3 vs. 3, ^d^ is > 6 vs. 6.

Figure 2 shows the distance covered in the different speed ranges. Significant differences were observed in relation to both the number of players and the type of marking. With the 3 *vs.* 3 format the distance covered at a speed of 0–6.9 km·h^−1^ was greater in games NMM (229.5 ±17.3 m in NMM *vs.* 198.9 ±27.9 m in MM, T_(18)_=2.94, *p*=0.009; ES=1.35). However, for this format the distance covered was greater during MM games for the speed ranges 7.0–12.9 km·h^−1^ (418.5 ±77.2 m in MM *vs.* 330.0 ±62.3 m in NMM, T_(18)_=2.82, *p*=0.011; ES =1.26) and 13.0–17.9 km·h^−1^ (111.8 ±41.7 m in MM *vs.* 70.4 ±39.5 m in NMM, T_(18)_=2.27, *p*=0.035; ES=1.02). In the 6 *vs.* 6 SGs, significant differences were only observed in the speed range 13.0–17.9 km·h^−1^, where the distance covered was greater when MM was used (141.7 ±39.6 m in MM *vs*. 108.4 ±28.0 m in NMM, T_(18)_=2.17, *p*=0.044; ES=0.98). With regard to the 9 *vs*. 9 SGs the results showed that when NMM was used, players covered a significantly greater distance at a speed of 13.0–17.9 km·h^−1^ than they did during 3 *vs.* 3 games (F_(2,25)_=9.87, *p*=0.001; ES=1.94). Similarly, during 9 *vs*. 9 games, players covered a significantly greater distance in the speed ranges 18.0–20.9 km·h^−1^ (F_(2,25)_=19.57, *p*=0.000) and >21 km·h^−1^ (F_(2,25)_=7.57, *p*=0.003), as compared with both the 6 *vs.* 6 (ES=1.39–1.66) and 3 *vs.* 3 games (ES=1.57–3.57). Finally, when MM was used during 9 *vs.* 9 SGs the distance covered at speeds >21 km·h^−1^ was significantly greater than during 6 *vs.* 6 games (F_(2,25)_=5.17, *p*=0.013; ES=0.91).

**Figure 3:**
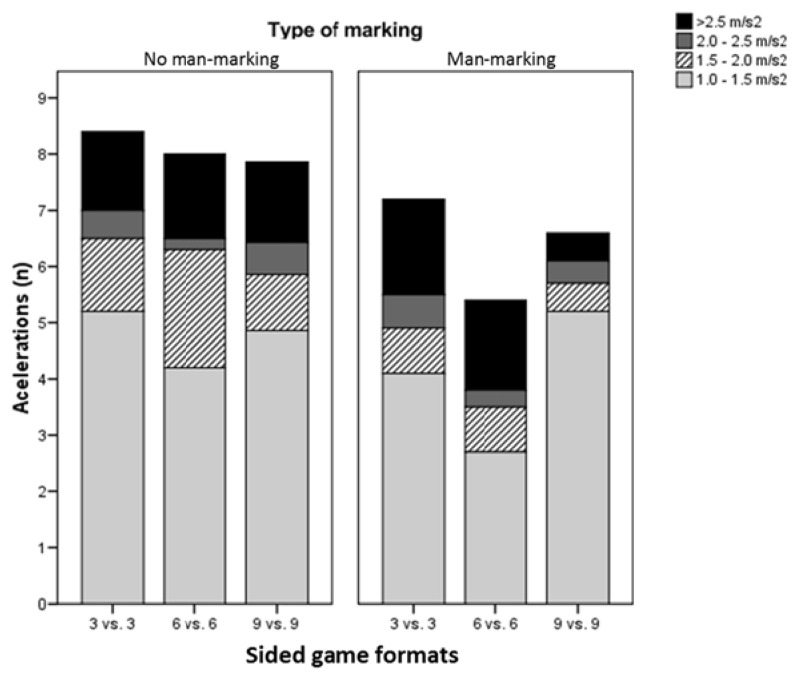
The number of accelerations (n) made in the established speed ranges for each of the SGs: 3 vs.3, 6 vs. 6, and 9 vs. 9.

Figure 3 shows the number of accelerations (n) made in the different intensity ranges for each of the SGs. This analysis revealed no significant differences in relation either to the number of players or the type of marking.

## Discussion

The aim of this study was to explore whether the type of marking (MM or NMM) and the number of players per team (3, 6, or 9) influenced the physical and physiological response of players in SGs. To the best of our knowledge, this is the first study to analyze the combined effect of these two variables during SGs. The main findings were that SGs involving MM were associated with greater physical demands, whereas physiological demands were greater in those SGs involving fewer players.

The only significant difference in physiological intensity that was related to the type of marking was observed in 6 *vs.* 6 SGs, where %HR_mean_ values were higher when NMM was used. The values of %HR_mean_ were similar to those obtained by Sampaio et al. (2007), who found no significant differences associated with the type of marking used in 2 *vs.* 2 and 3 *vs.* 3 SGs. It should be noted that our results are opposite to those reported by Ngo et al. (2012), who found that the %HR_res_ was higher in SGs with MM.

With respect to the number of players the value of the %HR_mean_ was significantly higher in 3 *vs.* 3 SGs (85.6%) than in 9 *vs.* 9 games (80%) when MM was used. When the data were pooled and analyzed regardless of the type of marking, the results showed a progressive increase in intensity as the number of players was reduced (9 *vs.* 9: 80.8%, 6 *vs.* 6: 83.7%, and 3 *vs.* 3: 84.1% of the HR_max_), although these differences were not statistically significant. Previous studies had observed similar results with higher intensity values in the HR during SGs with fewer players (Brandes et al., 2011; Castellano et al., 2013; Köklü et al., 2011). The reason for this could be a greater number of interventions of the players on the ball and opponents.

Significant differences were observed in the time spent by players in the different intensity ranges according to the number of players. In line with that, when pooling the data without considering the type of marking, the results showed that players spent longer at <80% HR_max_ during both the 6 *vs*. 6 and 9 *vs*. 9 SGs compared with the 3 *vs*. 3 games. This is consistent with previous studies that had also reported higher intensity levels in SGs involving fewer players (Aslan et al., 2013; Brandes et al., 2011; Hill-Haas, Dawson et al., 2009).

In the present study, physical demands (covered distance in metres) were greater in SGs that involved MM with the difference being significant in both the 3*vs.* 3 (736 m in MM *vs.* 634 m in NMM) and 6 *vs.* 6 formats (782 m in MM *vs.* 714 m in NMM). These results suggest that MM leads to increased physical demands as players have to follow their assigned opponent around the pitch. Furthermore, we also found that when they were required to MM, players covered greater distances at a speed of 13–17.9 km·h^−1^, this being the case for both the 3 *vs*. 3 (111 m in MM *vs.* 70 m in NMM) and 6 *vs.* 6 SGs (141 m in MM *vs.* 108 m in NMM). Since the opposing player always will try to shake off his marker, games in which players are required to MM imply greater physical intensity and the marker will cover more distance in moderate or high intensity zones, as opposed to just walking or standing still.

In relation to the global load indicators, we found that the player load was significantly greater when MM was used in the 3 *vs.* 3 SGs (95 *vs.* 82 AU), although no significant differences were observed in the 6 *vs.* 6 and 9 *vs.* 9 formats. Likewise, when MM was involved the work:rest ratio was significantly higher for 3*vs.*3 (3.4 *vs*. 2.3 AU) and 6 *vs.* 6 SGs (5.3 *vs.* 3.6 AU).

In general, the implementation of MM in SGs induced a higher physical demand (player load, work:rest ratio, total distance covered; distance covered at moderate-high speed). This is owed to the fact that players have to perform quicker movements and more running to receive passes potentially leading to greater exercise intensity (Ngo et al., 2012).

With respect to the number of players, the results showed that when NMM was required the total distance covered was significantly greater in 9 *vs.* 9 SGs (778 m) than in 3 *vs.* 3 games (634 m). This is consistent with previous studies (Brandes et al., 2011; Hill-Haas, Dawson et al., 2009) that had found that physical demands were greater when more players were involved. When we pooled and analyzed our data regardless of the type of marking, we again found that the distance covered by players during 3 *vs.* 3 games (685 m) was significantly less than the corresponding figure for 9 *vs.* 9 (762 m) and 6 *vs.* 6 SGs (748 m). Likewise, the maximum speed reached was greater during 9 *vs.* 9 SGs (19 km·h^−1^) than in 3 *vs.* 3 games (17.9 km·h^−1^).

Overall, these results indicate that physical demands increase in line with the number of players taking part in SGs. This is consistent with previous research (Brandes et al., 2011; Hill-Haas, Dawson et al., 2009), that had found higher mean distances and sprint duration in games involving more players. Basically, our findings are likely due to a greater overall playing area, which was increased in line with the number of players involved. As a result, players were provided greater scope for movement.

The analysis of accelerations, which should be considered a high-intensity activity (Varley et al., 2012), revealed no significant differences associated with the number of players or the type of marking. It is worth noting, however, that the number of low (1.0–1.5 m^.^s^2^) and medium intensity (1.5–2.0 m^.^s^2^) accelerations was higher when NMM was required, whereas the number of high-intensity accelerations (>2.0 m^.^s^2^) was greater in those games involving MM. The number of accelerations was also higher in the SGs with fewer players. Future research should seek to measure changes in acceleration during different kinds of SGs.

The main conclusion of this study is that it shows how variables such as the type of marking and number of players may be modified in order to alter the physical or physiological demands placed on amateur soccer players.

Specifically, games in which players were required to MM involved greater physical demands, and HR values were higher in SGs involving fewer players.

These findings could be used by coaches to achieve greater control over the load imposed on players, thereby enabling them to optimize their training regimens. When required to achieve high intensities with the aim of improving aerobic power, the coaches could use the MM rule or use fewer players per team. The use of MM or fewer players per team in phases of high training loads may be suggested.

## Figures and Tables

**Table 1:** Average values and standard deviation (±SD) in heart rate responses during various sided games

	SG	NMM	MM	Average
	
HR_mean_ (bpm)	3 *vs.* 3	162.8±7.0	168.6±8.9	165.7 ±8.4
	6 *vs.* 6	168.7±9.2	161.6±6.6	164.2 ±8.2
	9 *vs.* 9	161.1±11.0	157.7±12.8	159.2 ±11.8

	*Mean*	163.7±9.2	162.6±10.5	163.1 ±9.8

%HR_mean_ (%)	3 *vs.* 3	82.6±3.4	85.6±4.2^a^	84.1 ±4.0
	6 *vs.* 6	86.4±3.0^b^	82.0±3.2	83.7±3.8
	9 *vs.* 9	81.9±5.1	80.0±6.2	80.8±5.6

	*Mean*	83.3±4.2	82.5±5.1	82.9±4.7

HR_max_ (bpm)	3 *vs.* 3	180.1±7.3	182.5±8.2	181.3±7.6
	6 *vs.* 6	181.8±8.5	178.3±8.5	179.6±8.4
	9 *vs.* 9	175.0±7.3	175.4±12.1	175.2±10.0

	*Mean*	178.8±7.8	178.7±9.8	178.8±8.9

%HR_max_ (%)	3 *vs.* 3	91.4±3.6	92.6±4.3	92.0±3.9
	6 *vs.* 6	93.2±2.6	90.5±4.4	91.5±3.9
	9 *vs.* 9	88.9±3.1	89.0±5.8	88.9±4.7

	*Mean*	91.0±3.5	90.7±5.0	90.9±4.3

HR_mean_ is the mean heart rate, HR_max_ is the maximum heart rate, %HR_mean_ is the percentage mean heart rate relative to the individual maximum, %HR_max_ is the percentage maximum heart rate relative to the individual maximum, SG is a small-sided game, NMM is without man-marking, and MM is with man-marking.

a indicates significant difference with respect to 9 vs. 9;

b indicates significant difference with respect to MM (p<0.05 in both cases).

**Table 2:** Mean values and standard deviation (±SD D) for global load indicators during various sided games

	Players	NMM	MM	Mean
DT	3 *vs.* 3	634.5±64.5	736.5±86.6^c^	685.4±90.9
	6 *vs.* 6	714.8±77.6	782.7±52.5^c^	748.7±73.3^a^
	9 *vs.* 9	778.0± ±78.5^a^	750.2±70.5	762.6±73.3^a^

	*Mean*	704.1±91.9	756.5±71.5^c^	731.2±85.4

PL	3 *vs.* 3	82.1±11.4	95.1±11.8^c^	88.6±13.1
	6 *vs.* 6	91.9±12.8	95.5±6.2	93.7±9.9
	9 *vs.* 9	87.7±13.1	86.7±7.7	87.2±10.2

	*Mean*	87.2±12.7	92.4±9.6	89.9±11.4

V_max_	3 *vs.* 3	17.6±2.3	18.2±2.0	17.9±2.1
	6 *vs.* 6	18.9±2.0	18.4±0.8	18.7±1.5
	9 *vs.* 9	21.1± ±1.1^a^	20.3±1.5^a^	20.6±1.4^a^^b^

	*Mean*	19.0±2.3	19.0±1.7	19.0±2.4

WR	3 *vs.* 3	2.3±0.7	3.4±1.1^c^	2.9±1.1
	6 *vs.* 6	3.6±1.2	5.3±1.9^c^	4.4±1.8
	9 *vs.* 9	5.2±1.9	4.7±2.3	4.9±2.1

	*Mean*	3.5±1.7	4.4±1.9	4.0±1.9

DT is distance covered in m, PL is a player load in arbitrary units, V_max_ is maximum speed in km·h^−1^, WR is a work:rest ratio in arbitrary units in relation to the type of marking and the number of players in the SG, NMM is without man-marking and MM is with man-marking.

a indicates significant difference with respect to 3 vs. 3;

b indicates significant difference with respect to 6 vs. 6;

c indicate es significant differences with respect to NMM.
